# First *in vivo* analysis of the regulatory protein CP12 of the model cyanobacterium *Synechocystis* PCC 6803: Biotechnological implications

**DOI:** 10.3389/fpls.2022.999672

**Published:** 2022-09-13

**Authors:** Victoire Blanc-Garin, Théo Veaudor, Pierre Sétif, Brigitte Gontero, Stéphane D. Lemaire, Franck Chauvat, Corinne Cassier-Chauvat

**Affiliations:** ^1^CEA, CNRS, Institute for Integrative Biology of the Cell (I2BC), Université Paris-Saclay, Gif-sur-Yvette, France; ^2^Aix Marseille Univ, CNRS, BIP, UMR 7281, IMM, FR3479, 31 Chemin J. Aiguier, Marseille, France; ^3^Laboratoire de Biologie Computationnelle et Quantitative, CNRS, UMR7238, Institut de Biologie Paris-Seine, Sorbonne Université, Paris, France

**Keywords:** cyanobacteria, heterotrophy, carbon metabolism, redox regulation, photoproduction of terpenes, bisabolene, limonene

## Abstract

We report the first *in vivo* analysis of a canonical CP12 regulatory protein, namely the unique CP12 of the model cyanobacterium *Synechocystis* PCC 6803, which has the advantage of being able to grow photoautotrophically, photomixotrophically, and photoheterotrophically. The data showed that CP12 is dispensable to cell growth under standard (continuous) light and light/dark cycle, whereas it is essential for the catabolism of exogenously added glucose that normally sustains cell growth in absence of photosynthesis. Furthermore, to be active in glucose catabolism, CP12 requires its three conserved features: its AWD_VEEL motif and its two pairs of cysteine residues. Also interestingly, CP12 was found to regulate the redox equilibrium of NADPH, an activity involving its AWD_VEEL motif and its C-ter cysteine residues, but not its N-ter cysteine residues. This finding is important because NADPH powers up the methylerythritol 4-phosphate (MEP) pathway that synthesizes the geranyl-diphosphate (GPP) and farnesyl-diphosphate (FPP) metabolites, which can be transformed into high-value terpenes by recombinant cyanobacteria producing plant terpene synthase enzymes. Therefore, we have introduced into the Δ*cp12* mutant and the wild-type (control) strain our replicative plasmids directing the production of the monoterpene limonene and the sesquiterpene bisabolene. The photosynthetic production of both bisabolene and limonene appeared to be increased (more than two-fold) in the Δ*cp12* mutant as compared to the WT strain. Furthermore, the level of bisabolene production was also higher to those previously reported for various strains of *Synechocystis* PCC 6803 growing under standard (non-optimized) photoautotrophic conditions. Hence, the presently described Δ*cp12* strain with a healthy photoautotrophic growth and an increased capability to produce terpenes, is an attractive cell chassis for further gene manipulations aiming at engineering cyanobacteria for high-level photoproduction of terpenes.

## Introduction

Plants, algae, and cyanobacteria perform oxygenic photosynthesis to convert light energy to chemical energy (ATP and NADPH) that powers up the assimilation of CO_2_ by the Calvin–Benson–Bassham cycle (hereafter Calvin cycle) that is regulated by the intrinsically-disordered CP12 protein (for reviews on CP12 see Gontero and Maberly, 2012; Stanley et al., 2013; López-Calcagno et al., 2014; Gurrieri et al., 2022). CP12 is characterized by the AWD_VEEL core sequence and, in most cases, two N-terminal and C-terminal pairs of redox-sensitive cysteine (Cys-1 and Cys-2, and Cys-3 and Cys-4). In the dark, the oxidized cysteine pairs form disulfide bridges structuring two polypeptide loops required for the inactivation of two key enzymes of the Calvin cycle, phosphoribulokinase (PRK) and glyceraldehyde-3-phosphate dehydrogenase (GAPDH), by forming a GAPDH–CP12–PRK ternary complex. First, the C-terminal region of CP12 containing the distal cysteine disulfide bridge recruits GAPDH. Then, the N-terminal region of CP12 containing one cysteine pair followed by the consensus sequence binds PRK. In the eukaryotic alga *Chlamydomonas reinhardtii*, the core sequence of CP12 was found to act in the formation of the GAPDH–CP12–PRK complex and the protection of PRK from irreversible inactivation *in vitro* (Gérard et al., 2022). In the light, complex dissociation mediated by CP12 reduction *via* a thioredoxin allows GAPDH and PRK reactivation, and consequently CO_2_ fixation (Gurrieri et al., 2022).

All cyanobacteria possess at least one CP12 protein (Stanley et al., 2013), and many species also have one or several CP12-like proteins that are fused to an N-terminal cystathionine β-synthase (CBS) domain (Hackenberg et al., 2018). These CBS-CP12 proteins are also able to inhibit PRK but they do not form a ternary complex with PRK and GAPDH (Hackenberg et al., 2018).

Few typical CP12 proteins have been analyzed in cyanobacteria. The unique and canonical CP12 protein of the unicellular model *Synechocystis* PCC6803 (Ssl3364 in CyanoBase)^1^ was shown to form a 550-kDa-protein complex with PRK and GAPDH, which can be dissociated with NADPH. Complex reconstitution assays with heterologously-expressed proteins demonstrated that PRK and GAPDH bind to the N-terminal and the C-terminal parts of CP12, respectively. Indeed, a Cys-1 to serine mutant of CP12 was unable to bind PRK, whereas a Cys-4 to Ser mutant of CP12 lost the ability to bind GAPDH (Wedel and Soll, 1998). Similarly, the canonical CP12 protein of the unicellular cyanobacterium *Thermosynechococcus elongatus* can form a supramolecular complex with both PRK and GAPDH, the structure of which was solved recently (McFarlane et al., 2019).

The *Synechococcus* PCC 7942 CP12 variant lacking the two N-ter Cys residues (designated as Synpcc7942_0361 in CyanoBase)^2^ of canonical CP12 was also shown to form a 520 kDa protein complex with PRK and GAPDH, which can be dissociated by NADPH (Tamoi et al., 2005). A mutant strain lacking this two-Cys CP12 variant grew normally under standard light, but slower than wild-type (WT) cells under high light, or in a 12/12 h light/dark cycle (Tamoi et al., 2005; Tamoi and Shigeoka, 2021). Besides this two-Cys CP12 variant, *Synechococcus* PCC 7942 also has a four-Cys canonical CP12 protein (Synpcc7942_0252 in CyanoBase) which has not been studied yet.

As no canonical CP12 protein has been studied *in vivo* in cyanobacteria so far, we have analyzed the canonical four-Cys CP12 protein, i.e., Ssl3364 of *Synechocystis* PCC 6803. This model cyanobacterium was selected because (i) it has no other CP12 proteins which could have complicated our study, and (ii) it can grow not only photoautotrophically (light + CO_2_), but also photomixotrophically (low light + CO_2_ + glucose), or heterotrophically (glucose catabolism) when photosynthesis is inhibited by darkness, a knock-out mutation or the presence of 3-(3,4-dichlorophenyl)-1,1-dimethylurea (DCMU; Rippka et al., 1979; Veaudor et al., 2020).

We report that CP12 is dispensable for the photoautotrophic growth of *Synechocystis* PCC 6803, whereas it is essential for photoheterotrophic growth (catabolism of exogenous glucose). This CP12 activity requires its conserved AWD_VEEL motif and both its N-ter and C-ter pairs of cysteine residues. CP12 was also found to regulate the redox equilibrium of NADPH, an activity involving its AWD_VEEL motif and its C-ter cysteine residues, but not its N-ter cysteine residues. As NADPH powers up the synthesis of high-value terpenes by recombinant cyanobacteria producing heterologous terpene synthases, we tested the influence of CP12 on the production of limonene and bisabolene by our engineered strains of *Synechocystis* PCC 6803. Interestingly, the photosynthetic production of these two terpenes was higher (more than two-fold) in the Δ*cp12* mutant lacking CP12, as compared to the WT strain. Hence, the presently-described Δ*cp12* strain with a healthy photoautotrophic growth and an increased capability to produce terpenes is an attractive cell chassis for the future engineering of cyanobacteria for high-level photoproduction of terpenes, and possibly other high-value chemicals.

## Materials and methods

### Bacterial strains and growth conditions

*Escherichia coli* strains used for plasmid amplifications (TOP10 and NEB10 beta; Supplementary Table 1) or conjugative transfer of RSF1010-derived replicative plasmids (Supplementary Table 1) to *Synechocystis* (CM404; Mermet-Bouvier and Chauvat, 1994) were grown at 37°C (TOP10 and NEB10 beta) or 30°C (CM404) on LB medium containing appropriate antibiotic concentration: ampicillin (Ap) 100 μg.ml^−1^, kanamycin (Km) 50 μg.ml^−1^, streptomycin (Sm) 25 μg.ml^−1^ or spectinomycin (Sp) 75 μg.ml^−1^.

*Synechocystis* PCC 6803 (*Synechocystis*) was grown at 30°C, under continuous white light (2,500 lux; 31.25 μE m^−2^ s^−1^) or 8 h light/16 h dark cycles, and continuous agitation (140 rpm, Infors rotary shaker) in liquid MM mineral medium, i.e., BG11 (Stanier et al., 1971) enriched with 3.78 mM Na_2_CO_3_ (Domain et al., 2004) or MM*, i.e., MM supplemented with 17 μM Fe provided as green ferric ammonium citrate (the final concentration of Fe is 34 μM). For some experiments cells were grown under standard (2,500 lux; 31.25 μE m^−2^ s^−1^) or low (1,000 luxes; 12.5 μE m^−2^ s^−1^) lights, in the presence of glucose (5 or 55 mM) and/or 10 μM DCMU that inhibits photosynthesis (it blocks the photosystem II electron flow that normally reduces NADP^+^ to NADPH). Growth was monitored by regular measurements of optical density at 750 nm (OD_750_). In some cases, exponentially growing cells were serially diluted (five-fold) in MM, spread on glucose-containing MM solidified with 1% (10 g.l^−1^) Bacto Agar and incubated during 5–7 days at 30°C under specified light intensities.

Introduction in *Synechocystis* of DNA cassettes for targeted deletion and RSF1010-derived replicative plasmids were carried out using, respectively, natural transformation and conjugation (Supplementary Table 1) as previously described (Labarre et al., 1989; Mermet-Bouvier and Chauvat, 1994). Antibiotics used for selection were kanamycin (Km) 50–300 μg.ml^−1^, spectinomycin (Sp) 5 μg.ml^−1^, and streptomycin (Sm) 5 μg.ml^−1^.

### Construction of the Δ*cp12*::Km^r^ DNA cassette for targeted deletion and subsequent mutational analysis of the *cp12* gene

The two *Synechocystis* chromosomal DNA regions (about 300 bp in length each) flanking the *cp12* protein coding sequence (CS) were independently amplified by PCR, using specific oligonucleotides primers (Supplementary Table 2). They were then joined by standard PCR-driven overlap extension, in a single DNA segment harboring a *Sma*I restriction site in place of the *cp12* CS. After cloning in pGEM-T, the resulting plasmid (Supplementary Figure 1) was opened at its unique *Sma*I site where we cloned the transcription-terminator-less Km^r^ marker (a *Hinc*II segment from pUC4K plasmid) in the same orientation as the *cp12* CS it replaced (Supplementary Figure 1). The Δ*cp12*::Km^R^ DNA cassette was verified by PCR and nucleotide sequencing (Mix2Seq Kit, Eurofins Genomics). It was then transformed to *Synechocystis*, where homologous recombination occurring in the *cp12* flanking regions mediated the targeted replacement of *cp12* by the Km^R^ marker, in all copies of the *Synechocystis* chromosome (Supplementary Figure 2). The resulting Δ*cp12*::Km^R^ deletion mutant was then transformed by the *cp12_m_-Sm*^R^*/Sp*^R^ DNA cassettes for mutational analysis of the *cp12* gene (Supplementary Figures 3, 4). The *Sm*^R^*/Sp*^R^ transformants were analyzed by PCR and DNA sequencing to verify that they contained the *cp12_m_-Sm*^R^*/Sp*^R^ DNA cassettes integrated in place of the Δ*cp12*::Km^R^ locus, in all chromosome copies (Supplementary Figure 5).

### GAPDH and PRK enzymatic assays

Mid-log cultures harvested by centrifugation (10 min, 10^3^ g at room temperature) were quickly re-suspended in a buffer containing Tris–HCl 15 mM, EDTA 4 mM, NAD^+^ 0.1 mM, L-cysteine 2 mM, protease inhibitor cocktail 0.5 μg.ml^−1^, and 10% glycerol (pH 7.9). They were then frozen in a chilled (dry-ice/ethanol bath) Eaton press and broken (250 Mpa). Cell extracts were centrifuged, and supernatants were stored at −80°C until use. For each enzyme dosage, equivalent amounts of proteins were incubated 20 min at room temperature with or without 20 mM reduced/oxidized DTT. Assays were performed as previously described (Lebreton et al., 2003; Mekhalfi et al., 2012).

### Measurements of NAD(P)H fluorescence

Light-induced NADPH fluorescence were measured at 32°C with a Dual-PAM spectrophotometer (Walz, Effeltrich, Germany) equipped with the NADPH/9-AA module as described by one of us (Kauny and Sétif, 2014; Sétif et al., 2020). Exponentially growing *Synechocystis* cells washed twice and re-suspended in sterile MM medium at a concentration of 3.11–3.71 μg chlorophyll-a.ml^−1^ were incubated in darkness during 10 min in square 1 × 1 cm open cuvettes (Kauny and Sétif, 2014). The baseline fluorescence before illumination was arbitrarily set to zero. Then, cells were analyzed during several 1 min cycles of 0.5 s of illumination with an actinic light (λ_630nm_) of sufficient intensity (340 μmoles photons m^−2^ s^−1^) to trigger total photoproduction of NADPH (conditions where the Calvin cycle is not active; Kauny and Sétif, 2014; Sétif et al., 2020) followed by 55 s darkness to monitor NADPH decays.

### Terpene collection and quantification by gas chromatography–mass spectrometry

*Synechocystis* cells harboring the terpene production plasmids were photoautotrophically grown in the presence of selective antibiotics, in 125- or 250-ml erlenmeyers containing 25 or 50 ml cell suspensions overlaid with 20% (*v*/*v*) dodecane (analytical grade, Sigma-Aldrich) to trap terpenes (Lin and Pakrasi, 2019; Chenebault et al., 2020). At time intervals, 300 μl aliquots of the dodecane overlay were collected. 1 μl aliquots of these dodecane samples were injected in a split mode 10:1 (limonene) or 5:1 (E-α-bisabolene) into a GC–MS apparatus (Trace1300 (GC) + ISQ LT (MS), ThermoScientific). The quantification of the production of terpenes was performed as previously described (Chenebault et al., 2020).

## Results

### The unique CP12 protein of *Synechocystis* PCC 6803 is dispensable to cell growth under continuous light or light/dark regimes

**Figure 1:**
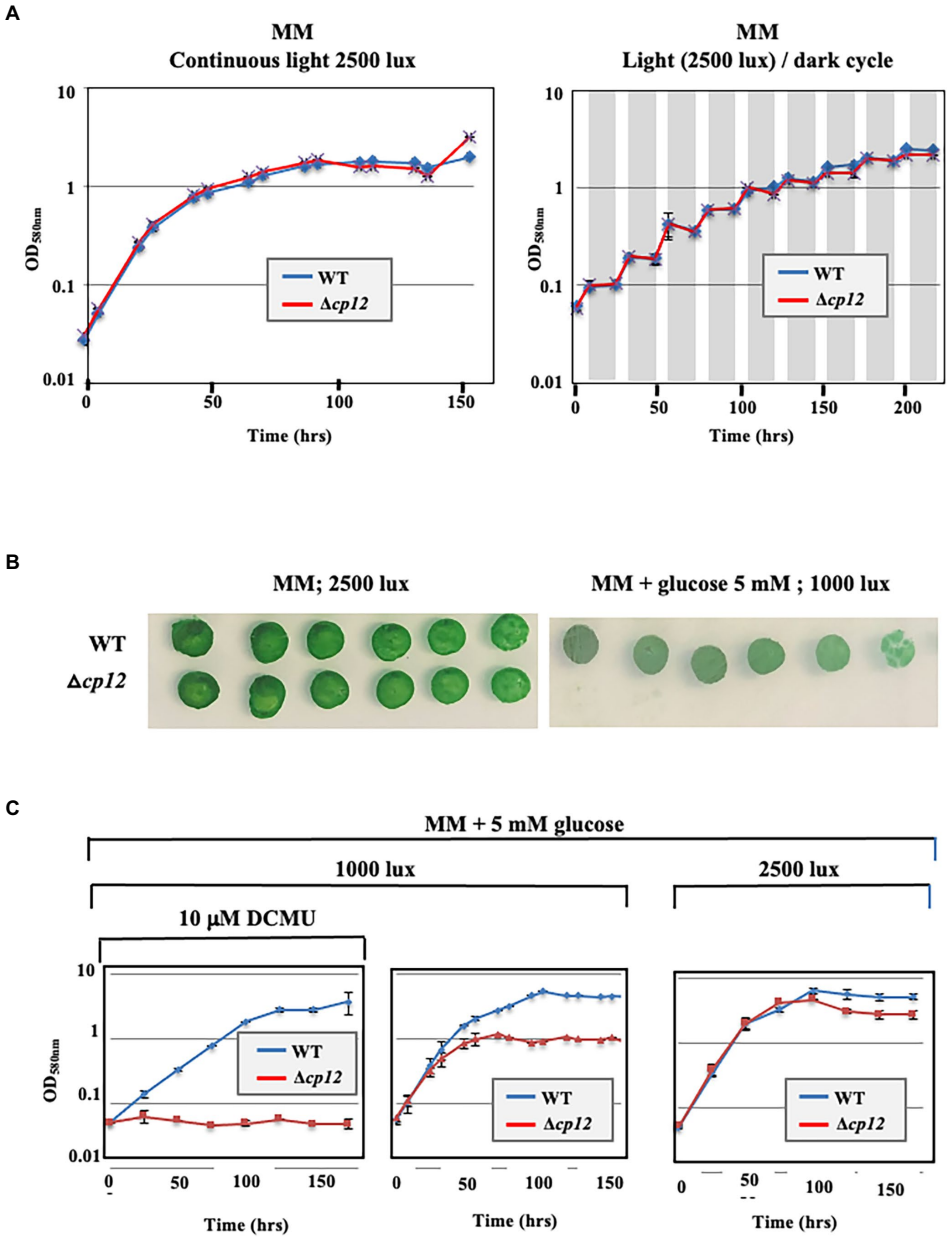
Influence of various conditions on the growth of the *Synechocystis* PCC 6803 WT strain and Δ*cp12* mutant. Typical growth legend of wild-type (WT) and deltacp12 mutant of *Synechocystis* PCC 6803 cultivated under either continuous light or a12 h light/12 h dark cycle (dark periods are shown in grey) in liquid **(A,C)** or solid MM media **(B)**.

To analyze *in vivo* the role of the CP12-encoding gene (*ssl1364* in CyanoBase), we constructed the Δ*cp12*::Km^R^ deletion cassette (see Materials and methods) to replace *cp12* by the Km^r^ marker. The resulting plasmid (Supplementary Figure 1) was introduced in *Synechocystis* by transformation (Labarre et al., 1989). A few Km^R^ transformants were analyzed by PCR with specific oligonucleotide primers (Supplementary Table 2) to verify that the Km^R^ marker gene had properly replaced *cp12* in some or all copies of the *Synechocystis* chromosome that is polyploid [it occurs at about ten copies per cell (Labarre et al., 1989)]. Indeed, the Δ*cp12*::Km^R^ mutant growing in standard photoautotrophic conditions in the presence of kanamycin possessed only Δ*cp12*::Km^R^ chromosome copies [Supplementary Figure 2, see the presence of the 1,421 bp PCR product characteristic of Δ*cp12*::Km^r^ chromosomes, and the absence of a 433 bp PCR band typical of WT (*cp12*^+^, Km^S^) chromosomes]. The absence of WT chromosome copies (*cp12*^+^, Km^S^) in the Δ*cp12*::Km^R^ mutant was confirmed by analyzing cells grown for multiple generations in absence of Km to stop counter-selecting the propagation of WT (*cp12*^+^, Km^S^) chromosome copies, which could have escaped PCR detection. As expected, the Δ*cp12*::Km^R^ mutant possessed only Δ*cp12*::Km^R^ chromosome copies (Supplementary Figure 2), and it grew as fit as the WT strain in both liquid and solid mineral media under standard light (Figures 1A,B). Together, these data demonstrate that the CP12 protein is not required for the standard photoautotrophic growth of *Synechocystis*.

**Figure 2:**
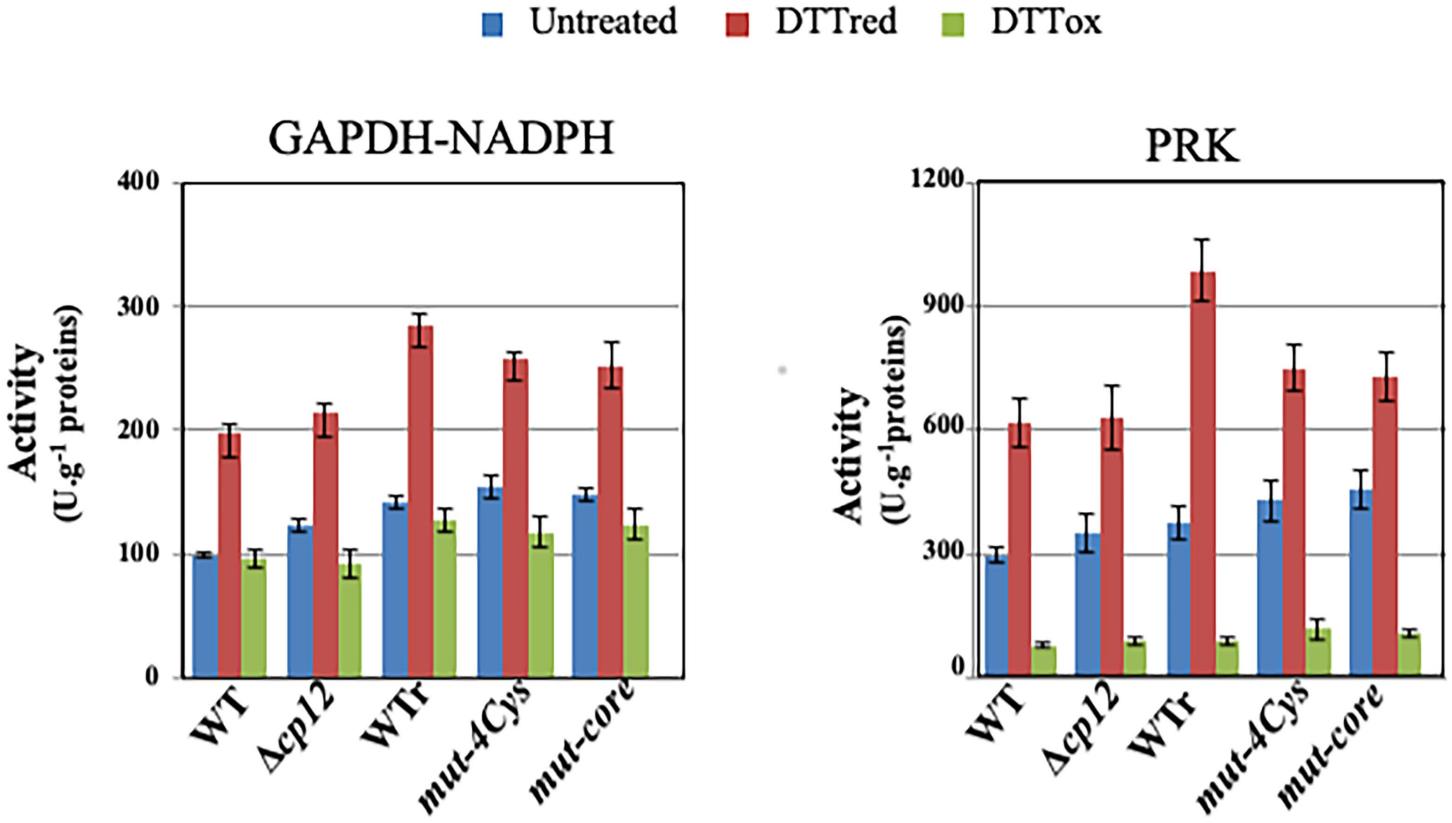
Influence of the CP12 protein on the activity of GAPDH and PRK enzymes. Histograms showing PRK and NADPH-dependent GAPDH activities in WT, WTr (WT rebuilt) and the indicated cp12 mutants grown under standard photoautotrophic conditions. Enzymatic assays were carried out on crude extracts treated or not with reduced DTT (DTTred) or oxidized DTT (DTTox) as described in material and methods. Data are expressed as the mean SD (*n* = 6).

Then we tested the influence of CP12 on the Calvin-cycle GAPDH and PRK enzymes, which play a prominent role in CO_2_ fixation. In WT cell extracts, both enzymes were activated by reduced DTT, while PRK and GAPDH were inhibited or not affected by oxidized DTT (Figure 2). Similar redox-dependent (sensitive to reduced or oxidized DTT) levels of GAPDH and PRK activities were observed in cell extracts from WT and Δ*cp12* strains (or site-directed *cp12* mutants presented below) growing photoautotrophically (Figure 2). These data are consistent with the findings that (i) the absence of CP12 did not alter the photoautotrophic growth of *Synechocystis* PCC 6803 (Figure 1) and (ii) CP12 does not interact with GAPDH and PRK (absence of GAPDH–CP12–PRK inhibitory complex) in photosynthetic organisms growing photoautotrophically (Gontero and Maberly, 2012; López-Calcagno et al., 2014; Gurrieri et al., 2022). Also interestingly, we found that CP12 is dispensable to *Synechocystis* PCC 6803 growing under light/dark (8/16 h) regime (Figure 1A). Similarly, the absence of the unique CP12 protein of the eukaryotic alga *C. reinhardtii* had no effect on its photoautotrophic growth under continuous light or light/cycle (Gérard et al., 2022).

### First evidence that CP12 plays an essential role in the catabolism of glucose that sustains the growth of *Synechocystis* PCC 6803 in absence of photosynthesis

**Figure 3:**
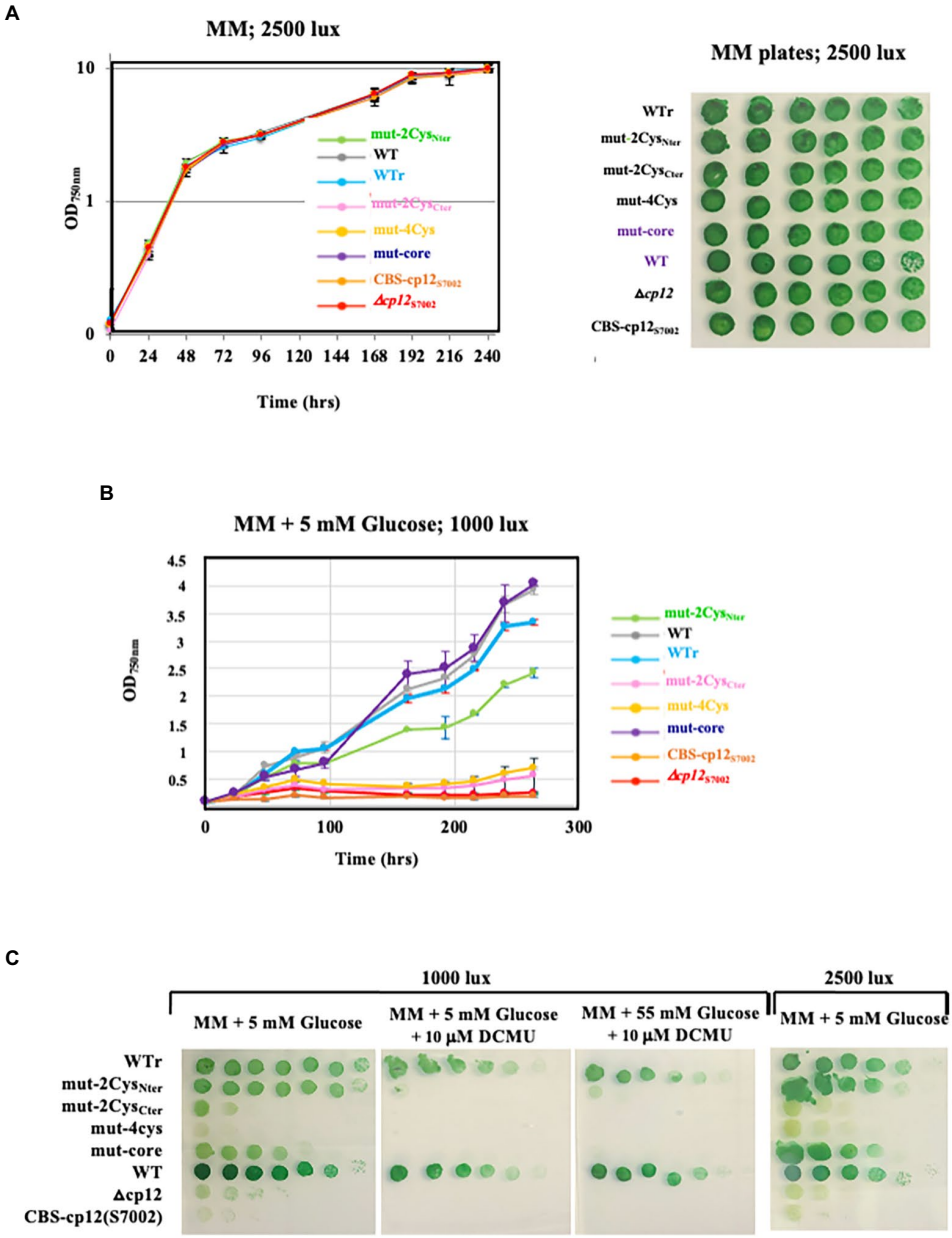
Influence of the WT and mutants CP12 proteins on the growth of *Synechocystis* PCC 6803 at the expense of glucose. Typical growth of WT and WT reconstructed (WTr) strains and the *cp12* mutants (Δ*cp12* △*cp12*, mut-4Cys, mut-2Cys_Nter_, mut-2Cys_Cter_, and mut-core) cultivated under continuous light of various intensities, in liquid **(A,B)** or solid media **(A,C)** containing or not the indicated concentrations of exogenous glucose and/or the photosynthesis inhibitor DCMU **(C)**.

*Synechocystis* PCC 6803 has the rare capability to compensate the absence of photosynthesis through intake and catabolism of exogenous glucose (Rippka et al., 1979), mainly performed through the oxidative pentose phosphate pathway (Yang et al., 2002; Narainsamy et al., 2013; Nakajima et al., 2014; You et al., 2015; Wan et al., 2017; Schulze et al., 2022). To better understand the influence of CP12 on the metabolism of *Synechocystis* PCC 6803, we have compared the growth of WT and Δ*cp12* cells cultivated at the expense of glucose to compensate for a decrease or complete loss of photosynthesis caused by low light or the addition of the photosynthesis inhibitor DCMU (Veaudor et al., 2020). While the WT strain grew well under photomixotrophic (light + CO_2_ + glucose) and photoheterotrophic (light + DCMU + glucose) conditions (Figures 1B,C, 3A–C), as expected (Veaudor et al., 2020), the Δ*cp12* mutant grew poorly under photomixotrophic conditions, and not at all under photoheterotrophic conditions (Figures 3B,C). These data showed that CP12 is required for the catabolism of glucose in *Synechocystis* PCC 6803.

### The essential role of CP12 in glucose catabolism involves its conserved C-ter and N-ter cysteine residues as well as its AWD_VEEL motif

Canonical CP12 proteins possess three conserved amino-acids (aa) parts: the AWD_VEEL conserved motif and, in most cases, two redox-sensitive cysteine pairs (N-terminal and C-terminal; Gontero and Maberly, 2012; Stanley et al., 2013; López-Calcagno et al., 2014; Gurrieri et al., 2022). The importance of these amino-acids for the *Synechocystis* PCC 6803 CP12-dependent glucose catabolism was presently analyzed by site-directed mutagenesis, as follows. Three *cp12* mutant genes were synthesized to replace the two N-ter cysteine residues and/or the two C-ter cysteine residues of CP12 by alanine residues (Supplementary Figures 3, 4). Another *cp12* mutant was synthesized to produce a CP12 protein where the charged aa of the core AWD_VEEL sequence were replaced by uncharged aa generating the AWa_VaaL mutant pattern (Supplementary Figures 3, 4). Each *cp12* mutant gene (*cp12_m_*) was assembled with an antibiotic-resistance marker gene (Sm^R^/Sp^R^) for selection, and subsequently cloned between the *cp12*-flanking chromosomal DNA regions to serve as platform of homology for genetic recombination that integrate each *cp12_m_-Sm*^R^*/Sp*^R^ DNA cassettes at the *cp12* locus, upon transformation to the Δ*cp12*::Km^R^ mutant. As a control, a similar *cp12_WT_-Sm*^R^*/Sp*^R^ DNA cassette was also constructed to re-introduce the WT *cp12* gene in the Δ*cp12*::Km^R^ mutant, thereby generating the WT reconstructed strain designated as WT_r_ (Supplementary Figures 3, 4).

As no cyanobacterial CBS-CP12 protein has been studied *in vivo*, we have also constructed the *cbs-cp1*2_S7002_*-Sm*^R^*/Sp*^R^ DNA cassette (Supplementary Figures 3, 4) to test whether the *Synechococcus* PCC 7002 CBS-CP12 protein (SYNPCC7002_A0199 in CyanoBase) can compensate the absence of the canonical CP12 protein of *Synechocystis* PCC 6803.

All five *Sm*^R^*/Sp*^R^ DNA cassettes were transformed to the Δ*cp12*::Km^R^ mutant, selecting for the Sm^r^/Sp^r^ and Km^S^ phenotype. In each case several transformant clones were analyzed by PCR and DNA sequencing (Supplementary Figure 5) to verify that they contained their CP12-encoding DNA cassette integrated in place of the Δ*cp12*::Km^R^ locus, in all copies of the polyploid *Synechocystis* chromosome. These reporter strains were named according to the nature of the CP12 protein they encode: WTr, mut-2Cys_Nter_, mut-2Cys_Cter_, mut-4Cys, mut-core, and CBS-CP12_S7002_ (Figure 3). They all grew as healthy as the true WT strain under standard photoautotrophic conditions (Figure 3A), in agreement with the above-mentioned finding that the absence of CP12 did not alter the photoautotrophic growth of *Synechocystis* PCC 6803 (Figures 1, 3A).

All CP12 reporter-strains were also incubated under photomixotrophic (light + CO_2_ + glucose) and photoheterotrophic (light + DCMU + glucose) conditions where cell growth is partially or totally sustained by the CP12-dependent catabolism of glucose, respectively. As expected, the reconstructed WTr control strain grew as fit as the true WT strain under both photomixotrophic and photoheterotrophic conditions (Figures 3B,C). In contrast, the Δ*cp12* mutant and the CBS-CP12_S7002_ reporter strain did not grow under these conditions (Figures 3B,C), showing that the *Synechococcus* PCC 7002 CBS-CP12 protein cannot compensate for the lack of the (canonical) CP12 protein of *Synechocystis* PCC 6803. This result is consistent with the finding that CBS-CP12 proteins are different from canonical CP12 proteins, for example in being unable to form a ternary complex with both PRK and GAPDH (Hackenberg et al., 2018).

Like the Δ*cp12* mutant, the mut-4Cys, and mut-2Cys_Cter_ mutants were unable to grow photomixotrophically and photoheterotrophically (Figures 3B,C), indicating that the CP12-dependent catabolism of glucose needs at least the two C-ter Cys residues of CP12. The other mutants mut-2Cys_Nter_ and mut-core grew well under photomixotrophic conditions, but not at all under photoheterotrophic conditions where the catabolism of exogenous glucose is crucial to compensate the absence of photosynthesis. Collectively, these data showed that the N-ter Cys pair and the AWD_VEEL motif are slightly less important than the C-ter Cys pair for CP12-dependent glucose catabolism.

### CP12 controls the redox equilibrium of NADPH, an activity involving its C-ter cysteine residues and AWD_VEEL motif, but not its N-ter Cys

Reduced nicotinamide adenine dinucleotide phosphate NAD(P)H is an essential electron donor in all organisms. In cyanobacteria, it provides the reducing power that drives the assimilation of inorganic nutrients and subsequent anabolic processes (Veaudor et al., 2020). Its fluorescence can be used for *in vivo* analysis of its formation (reduction of NAD(P) into NAD(P)H) and decay (oxidation of NAD(P)H), as shown by one of us (Kauny and Sétif, 2014; Sétif et al., 2020). These studies showed that while NADH and NADPH have identical fluorescence, most part of the light-dependent fluorescence signals can be attributed to NADPH.

Using this technique, also employed by many other groups (for examples see Assil-Companioni et al., 2020; Ogawa et al., 2021), we have analyzed *in vivo* the kinetics of light-induced NADPH formation (in conditions where the Calvin cycle is not active) and subsequent dark consumption in the WT strain and CP12 mutants. Practically, cells were exposed to several 1 min cycles of 0.5 s actinic light (λ_630nm_) of sufficient intensity (340 μmoles photons m^−2^ s^−1^) to trigger total photoproduction of NADPH (Kauny and Sétif, 2014; Sétif et al., 2020) followed by 55 s darkness to monitor NADPH decays. The results showed that the dark-induced decline of NADPH is faster in the Δ*cp12* mutant as compared to both the natural WT strain (strong difference) and the reconstituted WT strain (WTr, smaller difference), indicating that CP12 operate in NADPH-consuming pathway(s) other than the Calvin cycle.

**Figure 4:**
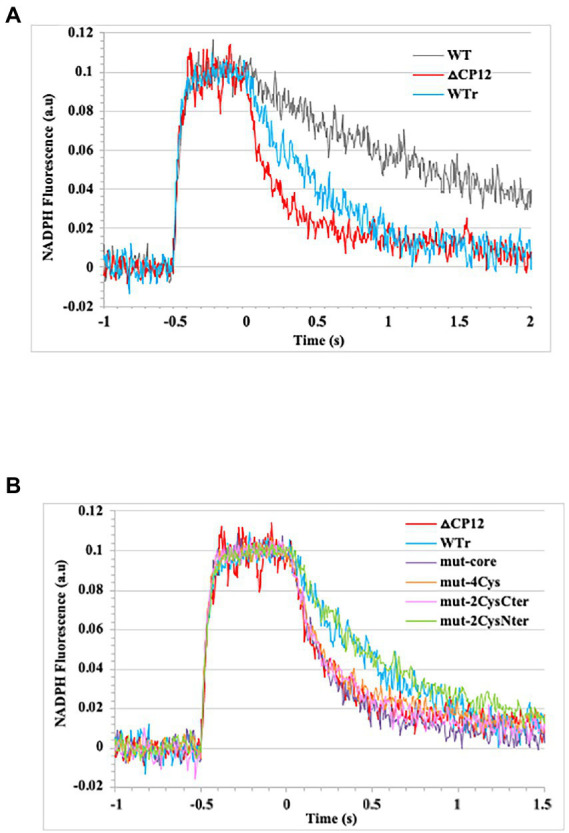
Kinetics of NADPH formation and decay measured *in vivo* by fluorescence. Cell suspensions were illuminated for 0.5 s with an actinic light (λ_630nm_) of sufficient intensity (340 μmoles photons m^−2^ s^−1^) to trigger total photoproduction of NADPH as described by one of us (Kauny and Sétif, 2014). Baseline fluorescence before the 0.5 s illumination was arbitrarily set to a value close to zero. Time 0 of NADPH decay was set at the end of the 0.5 s illumination period. An average of 40–135 of 1 min cycles of 0.5 s illumination (NADPH formation) and 55 s darkness (NADPH decay) were recorded. **(A)** Kinetics of NADPH formation and decay observed in the *Synechocystis* PCC 6803 strains WT, WTr (WT rebuilt) and △cp12. **(B)** Kinetics of NADPH formation and decay observed in the WTr strain, and the following *cp12* mutants: △*cp12*, mut-4Cys, mut-2Cys_Nter_, mut-2Cys_Cter_, and mut-core.

The difference between the dark-induced NADPH declines observed in the WT and WTr strains, which may result from a slightly different CP12 abundance, is not problematic because it is the WTr strain that served as the control strain for the analysis of the NADPH decline in the CP12 mutants. The NADPH decay was faster in the Δ*cp12* mutant than in the WTr strain (Figure 4A). Furthermore, the NADPH decline was as fast in the mut-core, mut-4Cys, and mut-2Cys_Cter_ mutants as in the Δ*cp12* mutant, whereas it was similarly slower in the mut-2Cys_Nter_ mutant and the WTr strain (Figure 4B). Altogether, these data indicate that CP12 acts in the redox equilibrium of NADPH, and that this CP12 activity requires its core consensus sequence and its C-ter cysteine residues, but not its N-ter cysteine residues.

### Deletion of the CP12-encoding gene in *Synechocystis* PCC 6803 engineered strains increases their production of limonene and bisabolene

**Figure 5:**
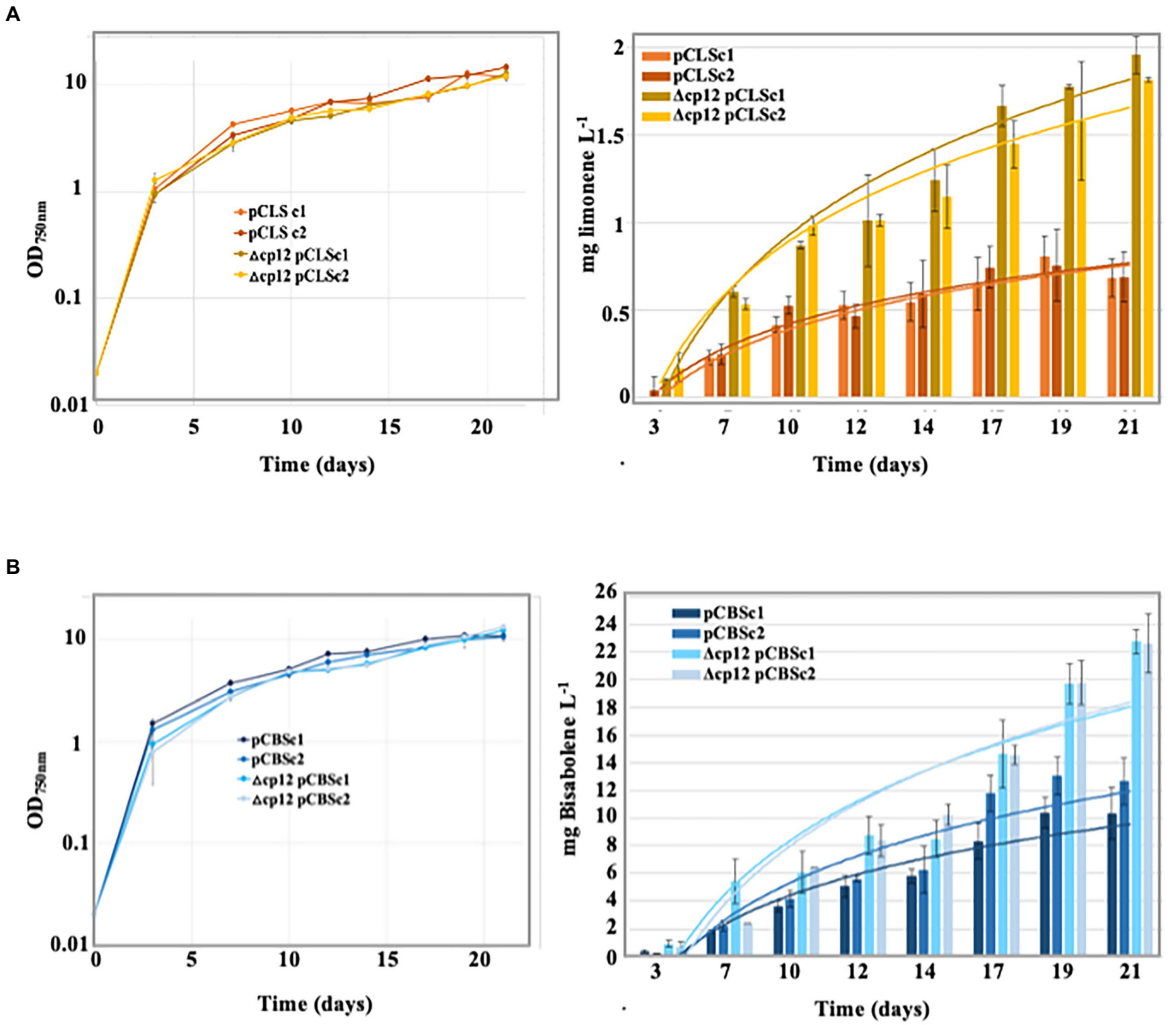
Growth and terpene production of the engineered *Synechocystis* PCC 6803 strains. Typical photoautotrophic growth [under a 20% (*v*/*v*) dodecane overlay] and terpene production of the *Synechocystis* strains expressing a terpene synthase gene from the following plasmids: **(A)** limonene synthase (pCLS) and **(B)** bisabolene synthase (pCBS).

We are interested in the engineering of cyanobacteria for the photosynthetic production of high-value compounds, such as the terpenes bisabolene and limonene which can be used to produce drugs, flavors, fragrances and biofuels (Lin and Pakrasi, 2019; Chenebault et al., 2020). *Synechocystis* PCC 6803 is well-suited for this purpose because it possesses the methylerythritol 4-phosphate (MEP) pathway that produces GPP (geranyl-diphosphate) and FPP (farnesyl-diphosphate), the metabolites that can be transformed into terpenes by heterologous (plant) terpene synthases produced in appropriate recombinant strains (Lin and Pakrasi, 2019; Cassier-Chauvat et al., 2021). As three MEP-pathway enzymes require NADPH (Lin and Pakrasi, 2019) whose level is dependent on CP12 (Figures 4A,B), we tested the possible influence of CP12 on terpene production. Therefore, we introduced in the Δ*cp12* mutant and the WT control strain our Sm^R^/Sp^R^ replicative plasmid vector pC for strong gene expression (Veaudor et al., 2018) and its derivatives producing the bisabolene synthase (pCBS) or the limonene synthase (pCLS; Chenebault et al., 2020). In each case, two independent Sm^R^/Sp^R^ clones were analyzed by PCR and DNA sequencing (Supplementary Figure 6) to verify that the pC, pCLS and pCBS plasmids replicated stably in *Synechocystis*. All these strains grew well under standard photoautotrophic conditions, irrespectively of the presence of a dodecane overlay used to trap terpenes (Figures 5A,B).

The production of limonene and bisabolene by the studied *Synechocystis* strains growing under standard photoautotrophic conditions were analyzed by GC–MS during 21 days, as we previously described (Chenebault et al., 2020). Neither limonene nor bisabolene were produced by the WT and Δ*cp12* negative control strains harboring or not the (empty) pC vector. In contrast, cells harboring pCBS and pCLS produced bisabolene and limonene, respectively. Interestingly, the levels of production directed by these plasmids were two-fold higher in the Δ*cp12* chassis as compared to the WT genetic background (Figures 5A,B). Hence, the Δ*cp12* strain with a healthy growth and an increased capability to produce terpenes seems to be an attractive cell chassis for work aiming at engineering a powerful cell factory for high-level photosynthetic production of terpenes.

## Discussion

The CP12 protein that regulates the CO_2_-fixing Calvin cycle pathway in photosynthetic organisms (cyanobacteria, algae and higher plants) contains a conserved AWD_VEEL amino-acids motif and, in most cases, two pairs (N- and C-terminal) of cysteine residues (for reviews on CP12 see Gontero and Maberly, 2012; López-Calcagno et al., 2014; Gurrieri et al., 2022). In darkness, the oxidized cysteine pairs of CP12 form disulfide bridges acting in the formation of the GAPDH–CP12–PRK complex that inhibits PRK and GAPDH. Under light, the GAPDH–CP12–PRK complex is dissociated by reduction *via* a thioredoxin, and CO_2_ fixation resumes (McFarlane et al., 2019; Gurrieri et al., 2022).

Every cyanobacteria possess at least one CP12 protein (Stanley et al., 2013), which had not been studied *in vivo*. Consequently, we have analyzed the single CP12 protein (canonical, four-Cys, Ssl3364 in CyanoBase) of *Synechocystis* PCC 6803, the unicellular cyanobacterium that is intensively studied for basic and applied science thanks to its powerful genetics (Cassier-Chauvat et al., 2021) and its capability to grow photoautotrophically (light + CO_2_), photomixotrophically (low light + CO_2_ + glucose) or heterotrophically (catabolism of exogenous glucose in absence of photosynthesis; Rippka et al., 1979; Veaudor et al., 2020). Using targeted gene deletion, site-directed mutagenesis and phenotypic analysis of the resulting mutants we showed for the first time that the unique CP12 protein of *Synechocystis* PCC 6803 is dispensable to cell growth under continuous light or light/dark regimes (Figure 1). Similarly, in the green alga *C. reinhardtii,* the cell growth under continuous light or light/dark regimes was also not affected in a mutant strain lacking the unique canonical CP12 (Gérard et al., 2022). A decrease in PRK activity level was observed in absence of CP12 (Gérard et al., 2022) as occurred in the higher plant *Arabidopsis thaliana* (López-Calcagno et al., 2017), whereas we did not observe any decrease in PRK activity (Figure 2). These findings suggest that different modes of CP12-dependent regulation occur in cyanobacteria, green algae and higher plants. Our results are also at variance with what was previously observed (Tamoi et al., 2005) in the distantly-related cyanobacterium *Synechococcus* PCC 7942 that has two CP12 proteins: (i) a four-Cys canonical CP12 (Synpcc7942_0252 in CyanoBase) and (ii) a two-Cys CP12 variant (Synpcc7942_0361) having only the C-terminal pair of Cys residues (Wedel and Soll, 1998; McFarlane et al., 2019; Gurrieri et al., 2022). A mutant *Synechococcus* PCC 7942 lacking this two-Cys CP12 variant grew normally under continuous light but slower than WT cells under a (12/12 h) light/dark cycle (Tamoi et al., 2005). It would thus be interesting to study the four-Cys canonical CP12 protein of *Synechococcus* PCC 7942.

Returning to the canonical (unique) CP12 protein of *Synechocystis* PCC 6803, we showed that it is essential for the catabolism of glucose that sustains cell growth in absence of photosynthesis (Figures 3B,C). All three CP12 conserved features, namely its N-ter and C-ter pairs of cysteine residues and AWD_VEEL motif are involved in the CP12-dependent catabolism of glucose (Figures 3B,C). Also interestingly, the CBS-CP12 protein of the unicellular cyanobacterium *Synechococcus* PCC 7002, which has an N-terminal CBS domain (Hackenberg et al., 2018), could not compensate for the lack of the canonical CP12 protein of *Synechocystis* PCC 6803 (Figures 3B,C). This finding indicates that CBS-CP12 and canonical CP12 proteins have different physiological roles as proposed (Stanley et al., 2013), in agreement with CBS-CP12 proteins being unable to form the ternary GAPDH-CP12-PRK complex observed with genuine cyanobacterial CP12 protein (Hackenberg et al., 2018).

The finding that the redox-sensitive CP12 protein of *Synechocystis* PCC 6803 operates in the metabolism (glucose catabolism) other than the Calvin cycle (CO_2_ fixation) prompted us to analyze, *in vivo*, the influence of CP12 on the redox equilibrium of NADPH. For this purpose we used the convenient method of measuring NADPH fluorescence developed by one of us (Kauny and Sétif, 2014; Sétif et al., 2020). The results indicated that CP12 acts on the dark induced oxidation of NADPH, and that this CP12 activity requires its core consensus sequence and its C-ter pair of cysteines, but not its N-ter pair of cysteines (Figure 4B).

Finally, it is known that *Synechocystis* PCC 6803 is widely-used for the photosynthetic production of high-value chemicals, such as terpenes that can be used for the production of drugs, flavors, fragrances and biofuels (Lin and Pakrasi, 2019; Chenebault et al., 2020). Indeed, *Synechocystis* PCC 6803 possesses the methylerythritol 4-phosphate (MEP) pathway that produces the GPP and FPP metabolites, which can be transformed into terpenes, after introduction and expression of heterologous genes encoding (plant) terpene synthase (Lin and Pakrasi, 2019; Cassier-Chauvat et al., 2021). As three MEP-pathway enzymes require NADPH (Lin and Pakrasi, 2019), the level of which is controlled by CP12 (Figures 4A,B), we have tested the influence of CP12 on terpene production. Therefore we have introduced our previously constructed replicative plasmids directing the production of the monoterpene (C_10_H_16_) limonene (pCLS vector; Chenebault et al., 2020) or the sesquiterpene (C_15_H_24_) bisabolene (pCBS plasmid) into the Δ*cp12* mutant and the WT strain. All reporter strains stably propagated their terpene-producing plasmid, and grew well under photoautotrophic conditions, in absence or presence of a dodecane overlay used to trap and measure terpenes (Figures 5A,B). Interestingly, the levels of bisabolene and limonene productions were two-fold higher in Δ*cp12* cells as compared to WT cells (Figures 5A,B).

These results indicate that the Δ*cp12* strain with a healthy photoautotrophic growth and an increased capability to produce terpenes is an attractive cell chassis for future gene manipulations aiming at engineering powerful cyanobacterial factories for high-level photoproduction of terpenes. Consistently, the levels of the photosynthetic production of bisabolene (22 mg.l^−1^ after 22 days) by our Δ*cp12*-pCBS strain is higher than what was reported for other pCBS-producing *Synechocystis* PCC 6803 growing under standard (non-optimized) photoautotrophic conditions 3.9 mg.l^−1^ after 7 days (Sebesta and Peebles, 2020) and 9.0 mg.l^−1^ after 12 days (Rodrigues and Lindberg, 2021).

## Data availability statement

The original contributions presented in the study are included in the article/Supplementary material, further inquiries can be directed to the corresponding author.

## Author contributions

CC-C and FC designed the work and wrote the manuscript with inputs from VB-G, TV, PS, BG, and SL. VB-G and TV carried out most of the experiments with the help of BG, SL, and PS. All authors contributed to the article and approved the submitted version.

## Funding

This work was supported in part by the CEA (program Focus ECC) and the ANR CalvinDesign project. VB-G and TV received a Ph.D. fellowship from the ANR and the CEA, respectively.

## Conflict of interest

The authors declare that the research was conducted in the absence of any commercial or financial relationships that could be construed as a potential conflict of interest.

## Publisher’s note

All claims expressed in this article are solely those of the authors and do not necessarily represent those of their affiliated organizations, or those of the publisher, the editors and the reviewers. Any product that may be evaluated in this article, or claim that may be made by its manufacturer, is not guaranteed or endorsed by the publisher.

## Footnotes

^1^ http://genome.microbedb.jp/cyanobase/

^2^ http://genome.microbedb.jp/cyanobase/
