# Supplementary material for: First in vivo analysis of the regulatory protein CP12 of the model cyanobacterium Synechocystis PCC 6803: Biotechnological implications
Source: Front Plant Sci. 2022 Sep 13;13:999672. doi: 10.3389/fpls.2022.999672 (PMC9514657; doi:10.3389/fpls.2022.999672)
Supplement: Supplementary file 1 [file Data_Sheet_1.PDF]

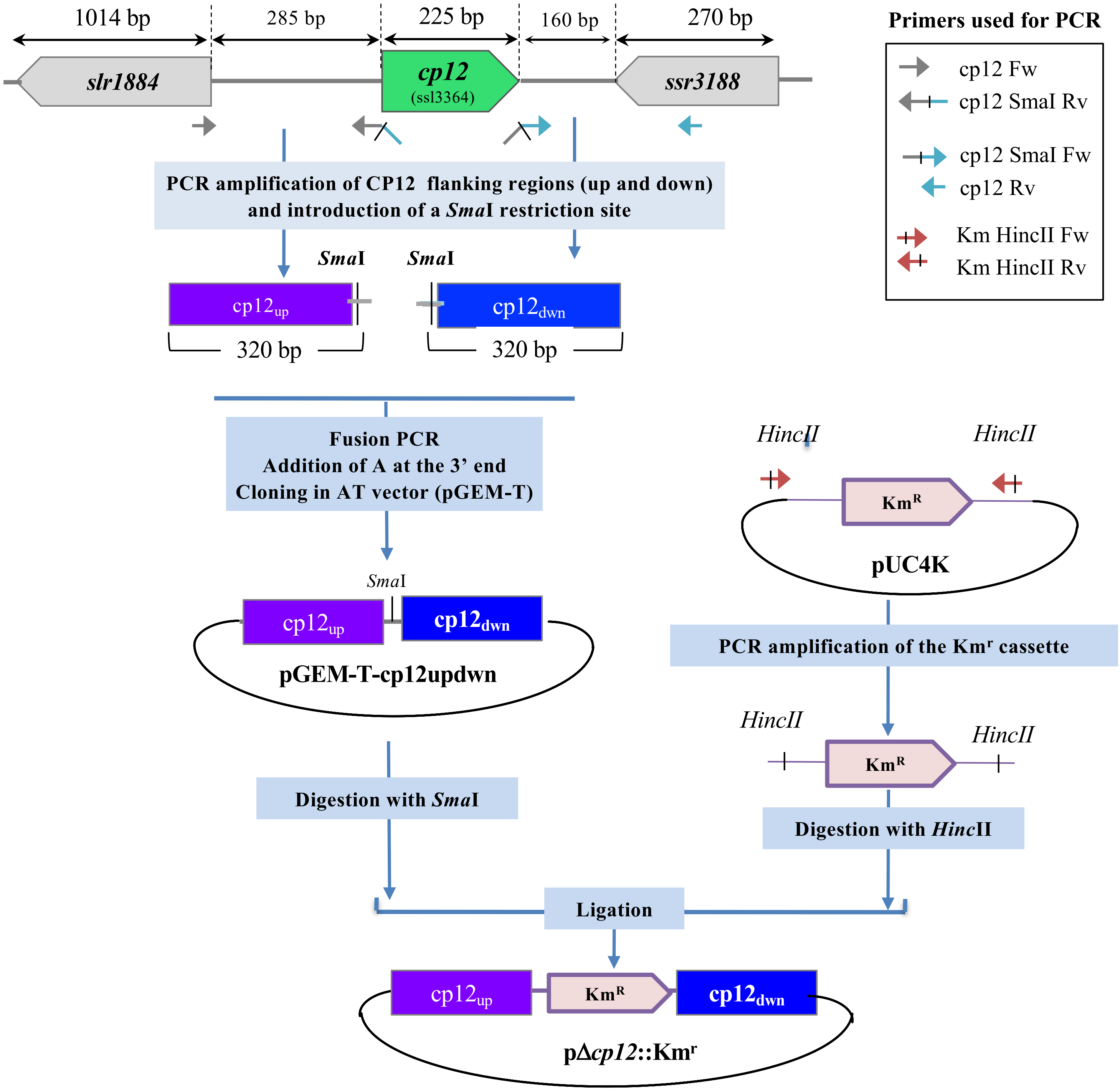

Supplementary Figure S1. Schematic representation of the *Synechocystis* PCC 6803 *cp12* chromosome locus and construction of the Δ*cp12*::Km^r DNA cassette for targeted deletion of the *cp12* gene. The genes are represented by large arrows respectively colored in green for *cp12* (also referred to as *ssl3364 in* CyanoBase), red for Km^R and grey (*cp12*-surrounding genes designated as *slr1884* and *ssr3188* in Cyanobase). For the sake of clarity, the *cp12*-flanking regions that served as platform of homology for DNA recombination mediating gene replacement are indicated by purple and blue rectangles, respectively.
