# Supplementary material for: First in vivo analysis of the regulatory protein CP12 of the model cyanobacterium Synechocystis PCC 6803: Biotechnological implications
Source: Front Plant Sci. 2022 Sep 13;13:999672. doi: 10.3389/fpls.2022.999672 (PMC9514657; doi:10.3389/fpls.2022.999672)
Supplement: Supplementary file 2 [file Data_Sheet_2.PDF]

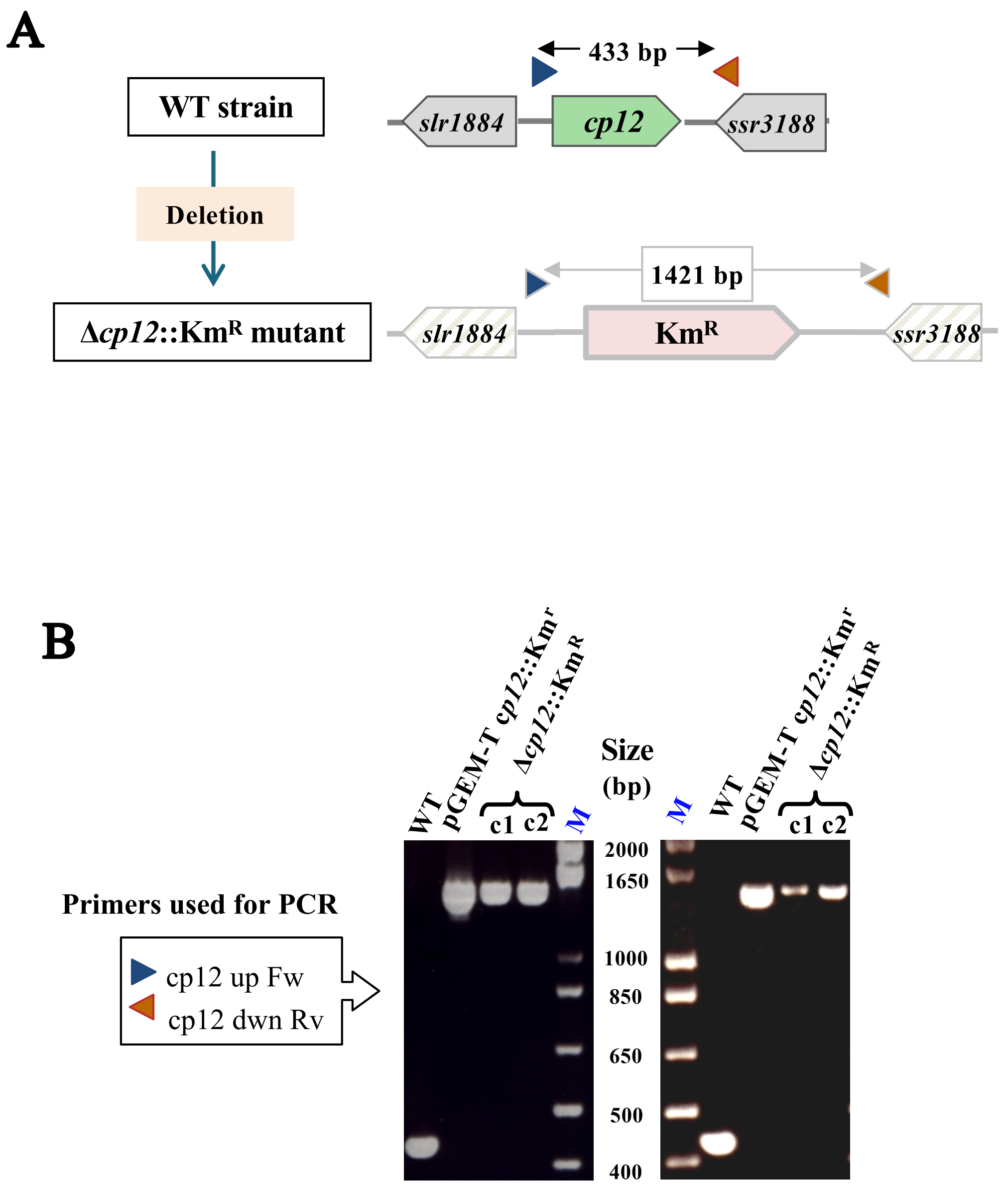

Supplementary Figure S2. PCR analysis of the CP12 chromosome loci in the *Synechocystis* PCC 6803 WT strain and D*cp12* mutants constructed in this study. (**A**) Genes are represented by arrows colored in green (*cp12*), grey (*cp12*-flanking genes) or pink (Km^R). PCR primers are shown as colored triangles and the size (bp) of resulting DNA products are indicated by double arrows. (**B**) Typical UV-light images of the agarose gels showing the relevant PCR products of two clones (noted as c1 and c2) of the Δ*cp12*::Km^R mutant grown in the presence (left gel) or absence (right gel) of Km. Note that they possess only Δ*cp12*::Km^R chromosome copies. Size marker (**M**) = 1 kb Plus DNA Ladder (Invitrogen).
