# Supplementary material for: First in vivo analysis of the regulatory protein CP12 of the model cyanobacterium Synechocystis PCC 6803: Biotechnological implications
Source: Front Plant Sci. 2022 Sep 13;13:999672. doi: 10.3389/fpls.2022.999672 (PMC9514657; doi:10.3389/fpls.2022.999672)
Supplement: Supplementary file 3 [file Data_Sheet_3.PDF]

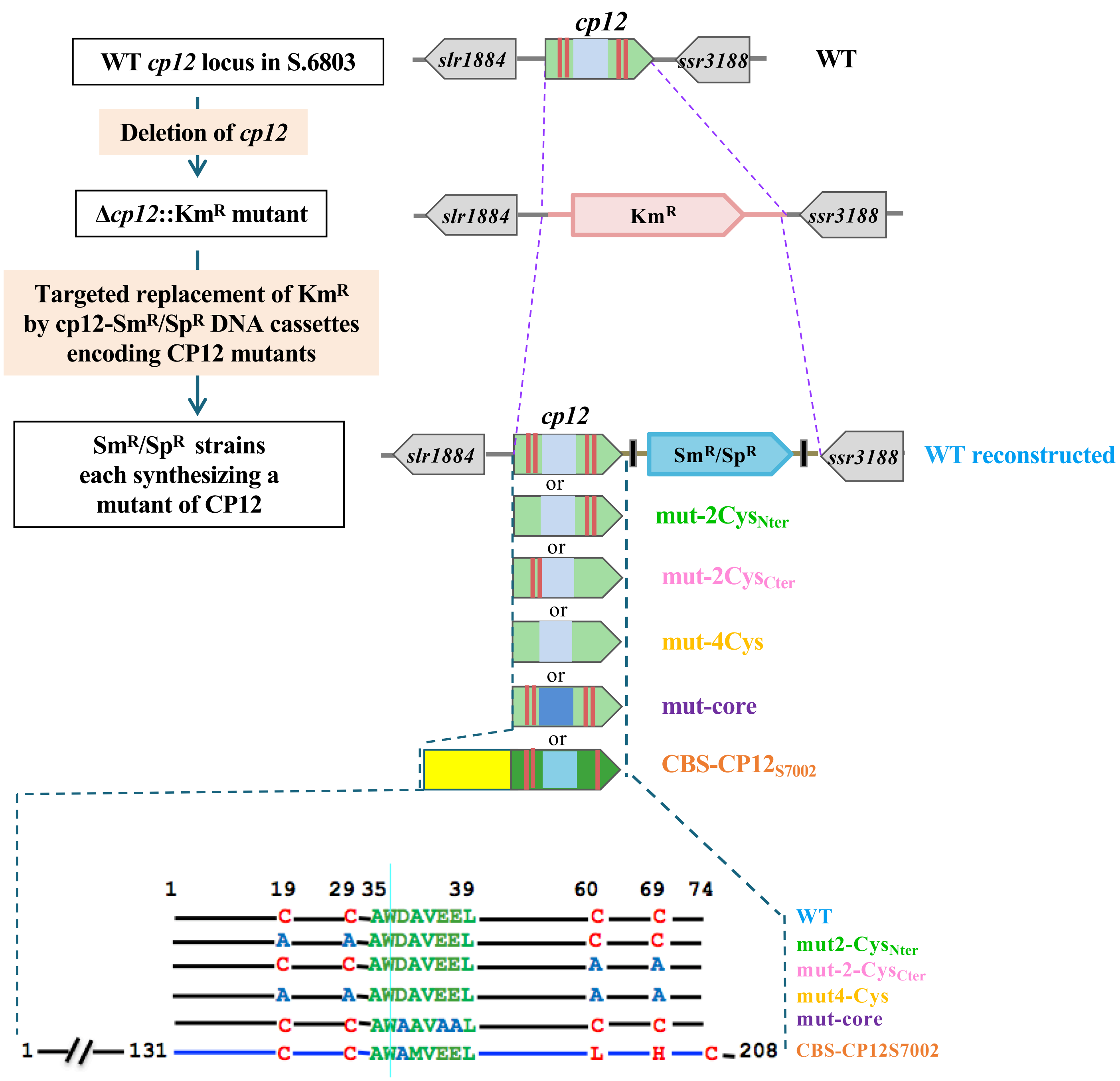

Supplementary Figure S3. Schematic representation of the gene-replacement strategy used for mutational analysis of the *Synechocystis* PCC 6803 *cp12* gene. Genes are represented by large arrows colored in green (*cp12*), grey (*cp12*-flanking genes), pink (Km^R) and Sm^R/Sp^R (blue, note that it is flanked by transcription/translation terminators shown as black vertical bars). The *Synechocystis* PCC 6803 *cp12* codons encoding the cysteine residues and the AWDAEEL core amino-acids (aa) are respectively indicated by red bars and blue squares (dark-blue when mutated). The aa residues are indicated according to the single letter code: A (alanine), C (cysteine), D (aspartic acid), E (glutamic acid), H (histidine), L (leucine) and W (tryptophan). In the *Synechococcus* PCC 7002 (S7002) *cbs-cp12* gene (SYNPCC7002_A0199 in Cyanobase) the parts encoding the 131 aa CBS (cystathionine β-synthase) domain and the CP12 domain are represented by yellow and dark-green rectangles, respectively.
