# Supplementary material for: First in vivo analysis of the regulatory protein CP12 of the model cyanobacterium Synechocystis PCC 6803: Biotechnological implications
Source: Front Plant Sci. 2022 Sep 13;13:999672. doi: 10.3389/fpls.2022.999672 (PMC9514657; doi:10.3389/fpls.2022.999672)
Supplement: Supplementary file 4 [file Data_Sheet_4.PDF]

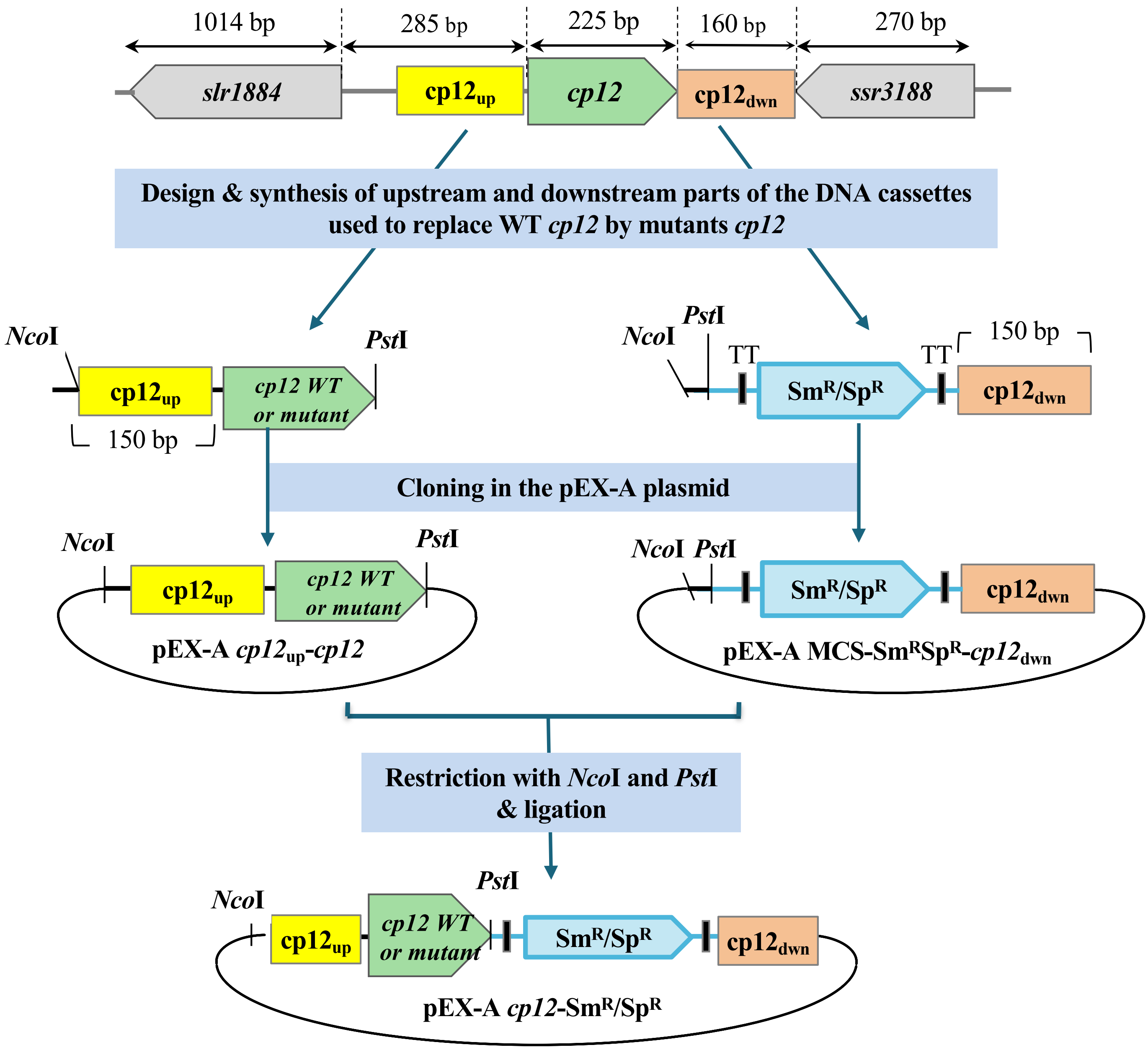

Supplementary Figure S4. Construction of the plasmid family for mutational analysis of the *Synechocystis* PCC 6803 *cp12* gene. Genes are represented by large arrows colored in green (*cp12* genes WT or mutants) or grey (*slr1884* and *ssr3188*), while regions flanking *cp12* are represented by yellow and orange rectangles, respectively. The Sm^R/Sp^R marker and its flanking transcription/translation terminators are represented by the blue arrow and the black vertical bars, respectively.
