# Supplementary material for: First in vivo analysis of the regulatory protein CP12 of the model cyanobacterium Synechocystis PCC 6803: Biotechnological implications
Source: Front Plant Sci. 2022 Sep 13;13:999672. doi: 10.3389/fpls.2022.999672 (PMC9514657; doi:10.3389/fpls.2022.999672)
Supplement: Supplementary file 5 [file Data_Sheet_5.PDF]

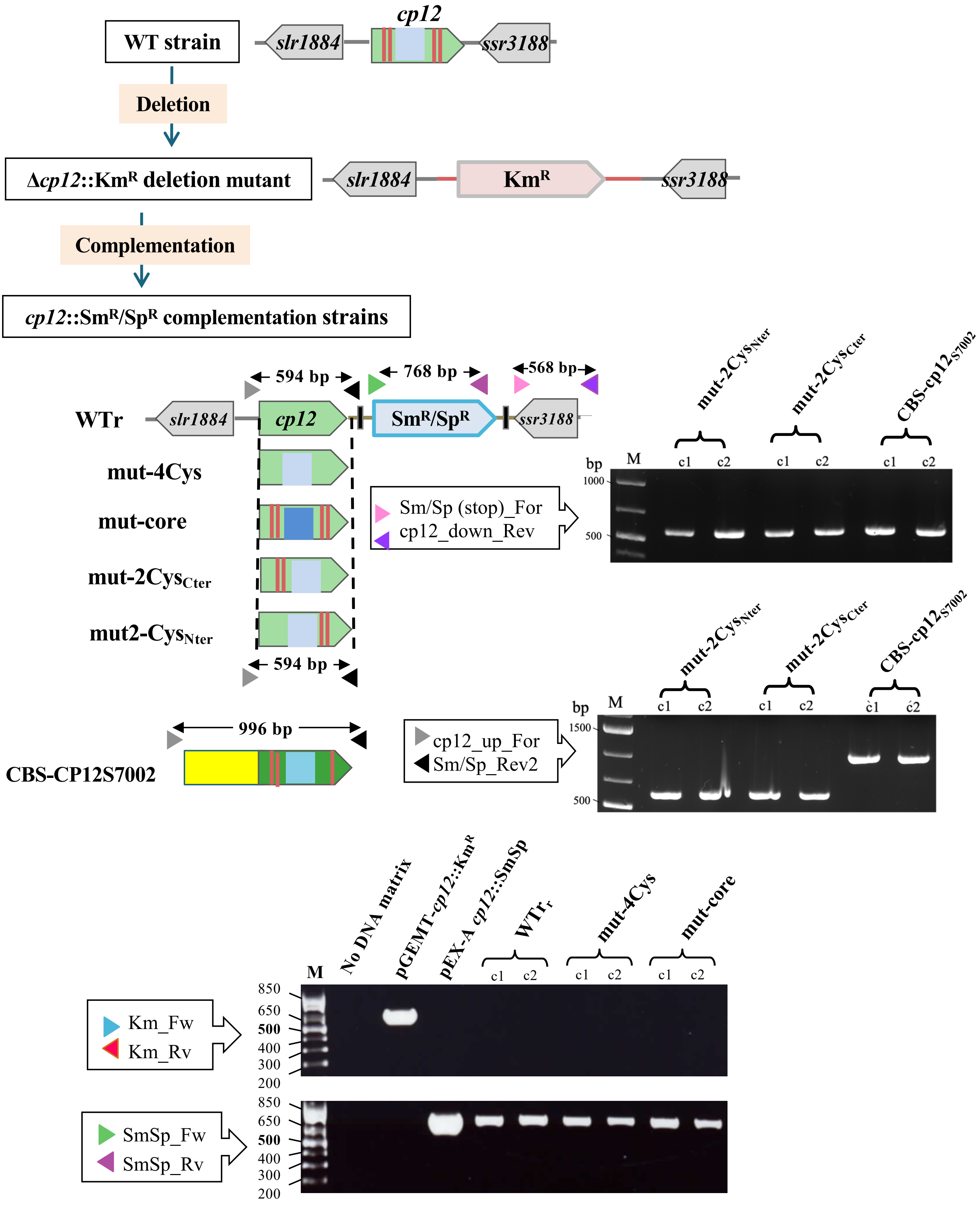

Supplementary Figure S5. PCR analysis of the *cp12* chromosome loci in the *Synechocystis* PCC 6803 WT strain and *cp12* mutants constructed in this study. Genes are represented by large colored arrows: green (*cp12*), grey (*cp12*-flanking genes), pink (Km^R marker) and blue (Sm^R/Sp^R marker flanked by transcription/translation terminators shown as black vertical bars). The *Synechocystis* PCC 6803 *cp12* cysteine residues and the AWDAEEL core amino-acids are indicated by red bars and blue squares (dark-blue when mutated), respectively. The CBS and CP12 domains of the *Synechococcus* PCC 7002 *cbs-cp12* gene are shown as yellow and dark-green rectangles. PCR primers and resulting DNA products are indicated by colored triangles and double arrows, along with the agarose gels used to analyze two clones (noted as c1 and c2) of each studied strain. Size marker (M) = 1 kb Plus DNA Ladder (Invitrogen).
