# Supplementary material for: First in vivo analysis of the regulatory protein CP12 of the model cyanobacterium Synechocystis PCC 6803: Biotechnological implications
Source: Front Plant Sci. 2022 Sep 13;13:999672. doi: 10.3389/fpls.2022.999672 (PMC9514657; doi:10.3389/fpls.2022.999672)
Supplement: Supplementary file 6 [file Data_Sheet_6.PDF]

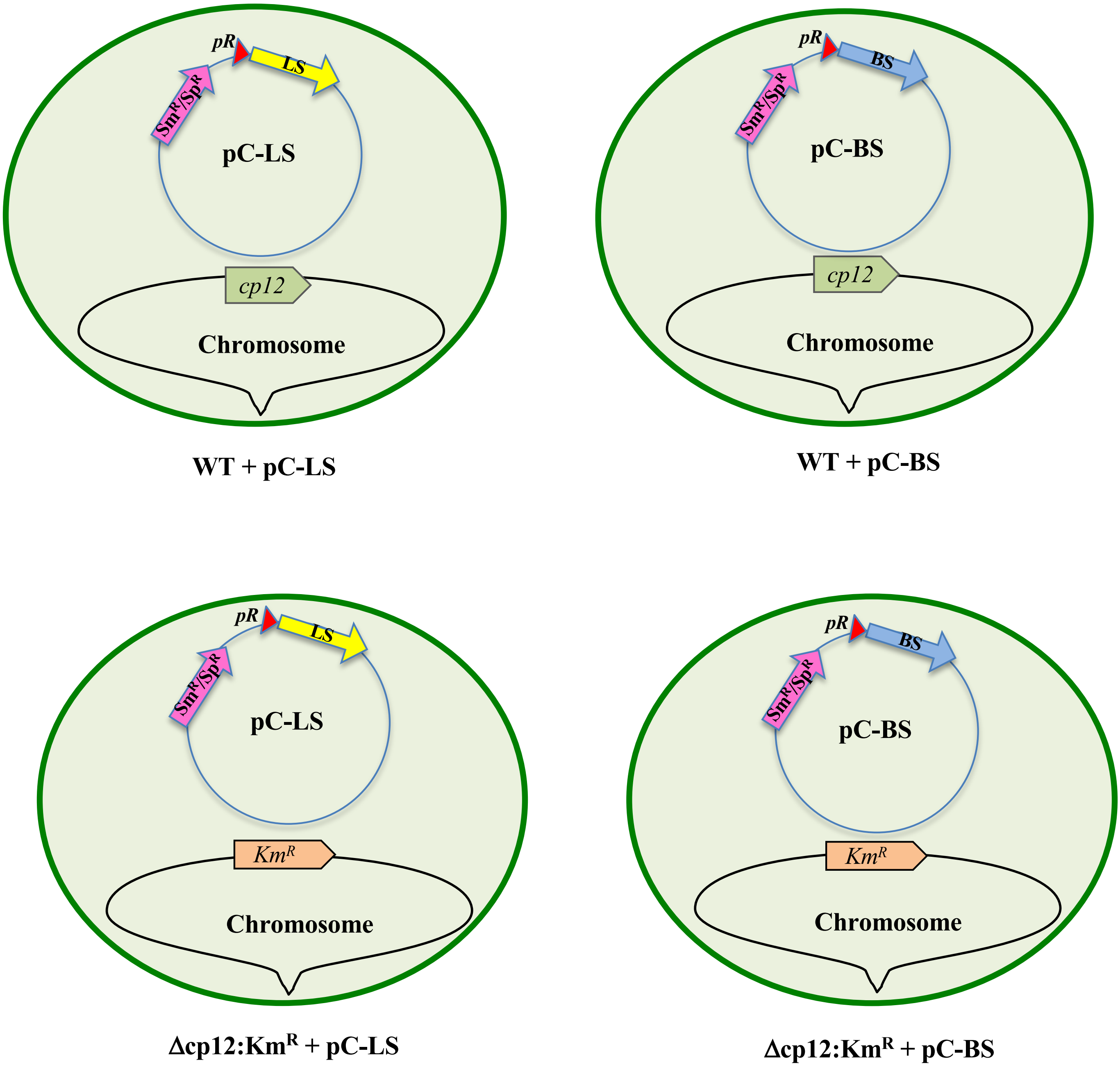

Supplementary Figure S6. Schematic representation of the terpenes producing *Synechocystis* PCC 6803 constructed in this study. Cells and their (polyploid) chromosome are represented by green and black oval shapes, respectively. The *cp12* gene present in WT cells and the Km^R marker replacing it in the Δ*cp12*::Km^R mutant are represented by green and orange arrows, respectively. The replicating plasmids over-expressing the genes encoding the limonene synthase (yellow arrow) or the bisabolene synthase (blue arrow) from the strong promoter *pR* (red triangle) are designated as pC-LS and pC-BS, respectively.
