# Supplementary material for: First in vivo analysis of the regulatory protein CP12 of the model cyanobacterium Synechocystis PCC 6803: Biotechnological implications
Source: Front Plant Sci. 2022 Sep 13;13:999672. doi: 10.3389/fpls.2022.999672 (PMC9514657; doi:10.3389/fpls.2022.999672)
Supplement: Supplementary file 7 [file Data_Sheet_7.PDF]

Supplementary Table S1 – Characteristics of the bacterial strains and plasmids used in this study

Strain/plasmid	Relevant features	Reference
***E. coli* and cyanobacterial strains**		
Escherichia coli CM404	*E. coli* strain harboring the self-transmissible plasmid pRK2013 enabling the conjugative transfer of RSF1010-derived plasmids into cyanobacteria	(Mermet-Bouvier and Chauvat, 1994)
Escherichia coli TOP10	*E. coli* strain for cloning and conjugation	Invitrogen
Escherichia coli NEB 10-beta	*E. coli* strain for cloning and conjugation	New England Biolabs
Synechocystis PCC 6803	Best-studied unicellular cyanobacterium holding a unique *cp12* gene encoding a canonical CP12 protein	Pasteur Institute (Paris, France)
**Cloning vectors and constructions of DNA cassettes**		
pGEM-T	AT-cloning vector with an Amp^r marker	Promega
pEX-A	Default subcloning vector for synthetic fragments with an Amp^r marker	Eurofins genomics
pUC4K	Vector used for PCR amplification of the Km^r cassette without TT	Pharmacia
pC	RSF1010-derived plasmid vector (Sp^R/Sm^R, Cm^R) harboring the strong p_R promoter for constitutive gene expression in *E. coli* and cyanobacteria	(Veaudor et al., 2018)
**Targeted deletions and integrations of *cp12* variants in *Synechocystis***		
pGEM-T *cp12*::Km^r **pΔcp12::Kmr**	pGEM-T with the Km^r marker flanked by the up- and downstream regions of the *cp12* (*ssl3364*) gene of *Synechocystis*. The construct allows complete replacement of the ORF by the Km^r cassette (+1 to +225 relative to the start codon)	This study Figure S1
pEX-A MCS-$SmSp^r$-$cp12_{dwn}$	pEX-A with the $SmSp^r$ + TT marker followed by the downstream region of the *cp12* gene of *Synechocystis* and preceded by *Nco*I and *Pst*I restriction sites. This MCS allows subcloning of other pEX-A constructs (*cp12* upstream region + mutated *cp12* gene) below.	This study
pEX-A	pEX-A with the WT *cp12* ORF preceded by the *cp12* upstream region and flanked by *Nco*I and *Pst*I	This study

cp12$_{up}$-cp12$_{WT}$	restriction sites.	
pEX-A cp12$_{up}$-cp12mut-4cys	pEX-A with the *cp12*mut-4cys ORF preceded by the *cp12* upstream region and flanked by *Nco*I and *Pst*I restriction sites.	This study
pEX-A cp12$_{up}$-cp12mut-2cys$_{Nter}$	pEX-A with the *cp12*mut-2cys$_{Nter}$ ORF preceded by the *cp12* upstream region and flanked by *Nco*I and *Pst*I restriction sites.	This study
pEX-A cp12$_{up}$-cp12mut-2cys$_{Cter}$	pEX-A with the *cp12*mut-2cys$_{Cter}$ ORF preceded by the *cp12* upstream region and flanked by *Nco*I and *Pst*I restriction sites.	This study
pEX-A cp12$_{up}$-cp12mut-core	pEX-A with the *cp12*mut-core ORF preceded by the *cp12* upstream region and flanked by *Nco*I and *Pst*I restriction sites.	This study
pEX-A cp12$_{up}$-CBS-cp12$_{S.7002}$	pEX-A with CBS-*cp12*$_{S.7002}$ ORF preceded by the *cp12* upstream region and flanked by *Nco*I and *Pst*I restriction sites.	This study
pEX-A cp12$_{WT}$::SmSp^r	pEX-A with the WT *cp12* ORF cloned after *Nco*I and *Pst*I digestion. This construct allows replacement of the Km^r marker of the Δ*cp12* mutant by the *cp12*$_{WT}$ gene followed by the SmSp^r cassette.	This study
pEX-A cp12mut-4cys::SmSp^r	pEX-A with the *cp12*mut-4cys ORF cloned after *Nco*I and *Pst*I digestion. This construct allows replacement of the Km^r marker of the Δ*cp12* mutant by a mutated *cp12* gene (Cys to Ala substitutions) and followed by the SmSp^r cassette.	This study
pEX-A cp12mut-2cys$_{Nter}$::SmSp^r	pEX-A with the *cp12*mut-2cys$_{Nter}$ ORF cloned after *Nco*I and *Pst*I digestion. This construct allows replacement of the Km^r marker of the Δ*cp12* mutant by a mutated *cp12* gene (Cys to Ala substitutions) and followed by the SmSp^r cassette.	This study
pEX-A cp12mut-2cys$_{Cter}$::SmSp^r	pEX-A with the *cp12*mut-2cys$_{Cter}$ ORF cloned after *Nco*I and *Pst*I digestion. This construct allows replacement of the Km^r marker of the Δ*cp12* mutant by a mutated *cp12* gene (Cys to Ala substitutions) and followed by the SmSp^r cassette.	This study
pEX-A cp12mut-core::SmSp^r	pEX-A with the *cp12*mut-core ORF cloned after *Nco*I and *Pst*I digestion. This construct allows replacement of the Km^r marker of the Δ*cp12* mutant by a mutated *cp12* gene (Asp and Glu to Ala substitutions) and followed by the SmSp^r cassette.	This study
pEX-A CBS-cp12$_{S.7002}$::SmSp^r	pEX-A with CBS-*cp12*$_{S.7002}$ ORF cloned after *Nco*I and *Pst*I digestion. This construct allows replacement of the Km^r marker of the Δ*cp12* mutant by the CBS-*cp12* gene of *Synechococcus sp.* PCC 7002 and followed by the SmSp^r cassette.	This study
**Replicative plasmids for strong constitutive expression of terpene synthase genes in *Synechocystis* WT and Δ*cp12* strains**		
**pCLS**	pC derived plasmid (Sp/Sm^R, Cm^S) harboring the LS (limonene synthase) encoding gene cloned	(Chenebault et al.,

	between the *Nde*I and *EcoR*I restriction sites	2020)
**pCBS**	pC derivative (Sp/Sm^R, Cm^S) carrying the BS (bisabolene synthase) encoding gene cloned between *Nde*I and *EcoR*I	(Blanc-Garin et al., 2022)

Abbreviations: CS, Protein Coding Sequence; Δ, deletion; mut, mutation; TT, transcriptional terminator; ORF, open reading frame; LS, limonene synthase; BS, bisabolene synthase.
