# Supplementary material for: First in vivo analysis of the regulatory protein CP12 of the model cyanobacterium Synechocystis PCC 6803: Biotechnological implications
Source: Front Plant Sci. 2022 Sep 13;13:999672. doi: 10.3389/fpls.2022.999672 (PMC9514657; doi:10.3389/fpls.2022.999672)
Supplement: Supplementary file 8 [file Data_Sheet_8.PDF]

Table S2. List of the PCR primers used in this study

Name	Sequence (5'→ 3')	Use and relevant feature(s)
**pEX Fw**	GGAGCAGACAAGCCCGTCAGG	Forward (Fw) and reverse (Rv) primers used for verification of the size and sequence of the DNA fragments cloned in the pEX plasmid's MCS.
**pEX Rv**	AGGCTTTACACTTTATGCTTCCGGC	
**cp12 Fw**	GGGCTGAACTCCAGAAAGGA	Forward (Fw) and reverse (Rv) primers used for PCR amplification of the *cp12* gene upstream region and *Sma*I restriction site addition.
**cp12 *Sma*I Rv**	CCATCAGCCC**CCCGGG**AAAGTTTAACCGCCT ACGATTGTCTACAGA	
**cp12 *Sma*I Fw**	GTTAAACTTT**CCCGGG**GGCTGATGGGGCAA ACCCAATGGCCGCAA	Forward (Fw) and reverse (Rv) primers used for PCR amplification of the *cp12* gene downstream region and *Sma*I restriction site addition.
**cp12 Rv**	GGCTACTCCATCAAGGGGAC	
**SP6 Fw**	TATTTAGGTGACACTATAG	Forward (Fw) and reverse (Rv) primers used for PCR amplification and DNA sequencing of sequences cloned in the pGEM-T vector.
**T7 Rv**	TAATACGACTCACTATAGGG	
**cp12 up Fw**	GGTCAGCAAAGTGAGGAAAA	Forward (Fw) and reverse (Rv) primers used to monitor the segregation of Δ*cp12*::Km^{r} chromosomes in *Synechocystis.*
**cp12 dwn Rv**	AGGTAATTGTCACTGCTTCA	
**Km Fw**	TCGAGGCCGCGATTAAATTC	Forward (Fw) and reverse (Rv) primers used to monitor the elimination and replacement of the Km^{r} marker by the *cp12*-Sm^{r}/Sp^{r} cassette in *Synechocystis* chromosomes.
**Km Rv**	GCGATTCCGACTCGTCCAAC	
**SmSp Fw**	AAGCGGTGATCGCCGAAGTA	Forward (Fw) and reverse (Rv) primers used to monitor the presence of the Sm^{r}/Sp^{r} marker in *Synechocystis* chromosomes.
**SmSp Rv**	TGGTGATCTCGCCTTTCACG	
**Km *Hinc*II Fw**	ACCTGCAGGGG**GTCGAC**GGAAAGCCAC	Forward (Fw) and reverse (Rv) primers used for Km^{r} cassette amplification and *Hinc*II restriction sites addition.
**Km *Hinc*II Rv**	GGCGCTGAG**GTCGAC**CTCGTGAAGAAG	
**Sm/Sp(stop)_For**	GCATTTGGTACAGCGCAG	Forward (For) and reverse (Rev2) primers used to verify the size and sequence of the downstream region of *cp12* variants in the *cp12*-Sm^{r}/Sp^{r} chromosomes of *Synechocystis.*
**Sm/Sp_Rev2**	GGCATCCAAGCAGCAAGC	

**LS_Rv3**	GATAGGCCCATGCGTTGAAGAT	Forward (Fw) and reverse (Rv) primers for PCR verification and DNA sequencing of the LS expression cassette from pCLS (introduced in *Synechocystis* strain by conjugation) enabling limonene production.
**LS_Fw3**	TGTCCGATTATAATGCCTCCG	
**BS_Fw2**	TGAGACCGACCAAGATAGC	Forward (Fw) and reverse (Rv) primers for PCR verification and DNA sequencing of the BS expression cassette from pCBS (introduced in *Synechocystis* strain by conjugation) enabling bisabolene production.
**BS_Fw3**	TAGGCATTGGAACGAGCG	
**BS_Fw4**	TATGACACCTACGGTACC	
**BS_Rv5**	CAAGAACTCAAGGCACTTC	
